# Integrative analysis indicates the prognostic value of circadian rhythm disruption in liver cancer: Potential for therapeutic targeting

**DOI:** 10.3389/fimmu.2022.1011264

**Published:** 2022-11-21

**Authors:** Rui-Qi Wang, Wei Cui, Jiayi Cai, Yihao Sun

**Affiliations:** ^1^ Department of Pharmacy, Zhuhai People’s Hospital, Zhuhai Hospital Affiliated with Jinan University, Jinan University, Zhuhai, Guangdong, China; ^2^ Department of Interventional Radiology, Guangdong Provincial People’s Hospital, Guangdong Academy of Medical Sciences, Guangzhou, Guangdong, China; ^3^ School of Stomatology, Zunyi Medical University, Zunyi, Guizhou, China; ^4^ Zhuhai Precision Medical Center, Zhuhai Interventional Medical Center, Zhuhai People’s Hospital (Zhuhai Hospital Affiliated with Jinan University), Zhuhai, China

**Keywords:** circadian rhythm disruption, liver cancer, single-cell transcriptomic analysis, prognostic factor, systematic analysis

## Abstract

Circadian rhythms regulate various biological processes, such as cell division and metabolism. Circadian rhythm disruption (CRD) is often associated with malignant tumor progression and poor prognosis. However, the effect of CRD on liver cancer prognosis has not been systematically analyzed or fully elucidated. Here, we developed a method to quantify and assess intratumoral CRD in a single-cell transcriptomic analysis of liver cancer and systematically analyzed the role of CRD in tumor progression and prognosis. Furthermore, a LASSO-Cox regression model based on 14 CRD genes was used to predict overall patient survival across multiple datasets. We found that malignant cells with high CRD scores were enriched in specific metabolic pathways, such as fatty acid metabolism and the trichloroacetic acid cycle. Intercellular communication analysis suggested that CRD regulates chemokine-mediated interactions. With the bulk transcriptomic datasets, we determined that LiverCRD scores were significantly correlated with macrophage infiltration levels and could guide targeted immunotherapy and chemotherapy strategies. In addition, LiverCRD is also associated with the mutational landscape—for example, TP53 mutation frequency was higher in high-CRD samples. Finally, the 14-gene-based LASSO-Cox regression model could accurately predict overall patient survival across datasets. In conclusion, Our proposed analysis reflects the relationship between CRD and the immune environment in liver cancer, suggesting that CRD may serve as a potential prognostic indicator. Our results may help guide targeted anti-tumor strategies.

## Introduction

Liver cancer is a global health threat, and its incidence is increasing worldwide (1). Hepatocellular carcinoma (HCC) is the most common type of liver cancer, and hepatitis B virus (HBV) and hepatitis C virus (HCV) infections (2) are key pathogenic factors in the development of HCC. Although our understanding of HCC pathophysiology has improved, this knowledge has not yet been translated into clinical practice. The dominant mutational drivers of HCC, such as TERT, TP53, and CTNNB1, are difficult to target (3, 4). In addition, the efficacy of translating molecular and immune classifications into biomarkers to guide therapy remains under investigation. Molecular subtypes of HCC have been defined according to the primary molecular drivers and pathways or the immune status of the tumor (5–7). The proliferative class accounts for approximately 50% of HCC cases; it is enriched in TP53 mutations and FGF19 or CCND1 amplification and has the worst prognosis. The non-proliferative tumor category accounts for the remaining 50% of HCC cases, is more prevalent in alcohol-related and HCV-related HCC, and has a better prognosis. Our understanding of the molecular features of HCC has been further improved *via* the classification of HCC according to immune cell status: immune-activated, immune-exhausted, immune-intermediate, and immune-excluded (8). Immune-activated HCC (found in 20% of cases) exhibits high active helper T (CD4+) and cytotoxic T (CD8+) cell infiltration and is responsive to immune checkpoint inhibitor (ICI) therapy. In contrast, immune-exhausted tumors are dominated by TGF-β driven CD8(+) cell depletion. Immuno-excluded tumors, representing the other end of the spectrum, are characterized by a lack of T-cell infiltration, an increase in regulatory T cell numbers, classical Wnt signaling, and other immunosuppressive cascades. Immune-excluded tumors are predominantly resistant to ICI therapy.

Circadian rhythm disruptions (CRDs) can lead to metabolic and immune diseases (9, 10). In addition, the expression of core clock genes and proteins is significantly attenuated in highly malignant or aggressive tumors, suggesting that circadian rhythms may be associated with cell differentiation (11). The circadian clock alters tumor cell metabolism and controls tumor development by interacting with non-clock transcription factors (9, 12), ultimately affecting cell differentiation and proliferation. On a molecular level, the biological clock is based on a transcription–translation feedback loop (13). Multiple transcription factors, nuclear receptors, and coregulators bind to specific DNA recognition sequences, such as E-boxes, retinoic acid receptor-related orphan receptor response elements, and D-boxes, which engage in driving biological periodicity. The circadian rhythm of tumor cells appears to be somewhat disturbed compared to that of normal tissue. Meta-analysis has revealed widespread misexpression of clock genes in multiple human cancers (14). Notably, the abnormal expression of clock genes appears to correlate strongly with the stage or aggressiveness of various cancers. For example, the biological clock is disrupted in advanced stages of Hodgkin’s lymphoma (15). Clock genes are also more dysregulated in triple-negative breast cancers than in other breast cancers (16). Moreover, the circadian clock and cell differentiation are closely linked. The core clock is not clearly expressed in undifferentiated pluripotent stem cells, but it gradually manifests with cell differentiation and biological development (17). However, the molecular function and mechanism of circadian rhythm disruption in liver cancer have not yet been elucidated. Therefore, it is necessary to systematically analyze the relationship between circadian rhythm disruption and tumor progression in liver cancer.

In this study, we employed a computational method to calculate and assess intratumoral CRD in liver cancer cells using a single-cell transcriptomic dataset. CRD scores predicted tumor responses to immune checkpoint blockade (ICB) therapy and can guide targeted strategies for chemotherapy. Furthermore, a LASSO-Cox regression model based on 14 CRD genes was used to predict overall patient survival across multiple datasets. Through systematic analysis, we aimed to determine the role of circadian rhythm disruption in tumor progression and prognosis. Targeted drug prediction analysis can provide new therapeutic strategies for combination antitumor therapy.

## Materials and methods

### Datasets

In this study, we analyzed two publicly available single-cell transcriptome datasets on liver cancer annotated with clinical information (18). We integrated and normalized the two datasets according to a previously reported pipeline (19). The merged single-cell transcriptome data were log-normalized and scaled for downstream analysis using Seurat V4.0.1. In addition to single-cell transcriptome data, we downloaded multiple bulk liver cancer transcriptome datasets and their corresponding clinical information to verify results obtained from the merged single-cell transcriptome datasets. The log2 normalized and transformed gene expression matrix of the Cancer Genome Atlas Liver Hepatocellular Carcinoma (TCGA-LIHC) dataset was downloaded from the University of California, Santa Cruz (UCSC) Xena browser (https://xenabrowser.net/). Transcriptomic and clinical data from 159 pairs of tumor and normal samples taken from Chinese HCC patients with HBV infection were downloaded from the National Omics Data Encyclopedia database (https://www.biosino.org/node/) using the accession number OEP000321 (20). The LIRI-JP dataset, including transcriptome data from 231 HCC samples, was downloaded from the International Cancer Genome Consortium Data Portal (https://dcc.icgc.org/). Gene expression microarray data and detailed clinical information on GSE14520 (21) (including 221 HCC samples) were downloaded from the Gene Expression Omnibus database (https://www.ncbi.nlm.nih.gov/geo/).

### Liver CRD score calculation

CRD-related genes (CRGs) were collected from the CircaDB database (22). We employed gene set enrichment analysis to infer the CRD level of each cell based on the CRG expression matrix. First, we normalized all the sample matrices to minimize the effect of outliers. The relative ranking of CRDs among all genes reflects the overall CRD status of the sample. We used single-sample gene set enrichment analysis (ssGSEA) to calculate the relative CRD enrichment score of each sample (LiverCRD score), which represented the CRD status of the sample. We then employed a random sampling strategy to determine the abnormal gene set activity score. All genes were divided into 50 expression bins based on their average expression. The number of occurrences of CRGs, that is, their frequency within each bin, was calculated and identified as N. Based on random sample N timings, random signature genes from each bin were chosen. In other words, the total number of random signature genes K matched the number of CRGs. This process was repeated 1000 times at random. We then created a random score as the mean of the K x 1000 random signatures sampled to define the background level of CRGs. The threshold for determining abnormal CRD levels was set at 75% based on quartiles, which was -0.24.

### Weighted correlation network analysis

We performed a weighted correlation network analysis (WGCNA) (23) to identify CRGs significantly associated with one or more clinical properties of the samples. This analysis was performed using the R package WGCNA V1.70-3. We screened the gene modules that were significantly positively correlated with the tumor stage (p< 0.05). Finally, 206 genes were screened for subsequent analysis and modelling.

### Intercellular communication analysis

We used data obtained from CellphoneDB (24) to analyze intercellular interactions. First, we investigated whether there was significant interaction (p< 0.05) between the two cell types (tumor and normal) by evaluating the average expression levels of annotated ligand-receptor pairs in the STRING database (25). Ligand-receptor pairs associated with p< 0.05 were subsequently screened to evaluate the relationship between the two cell types. Given that cytokines are critical for intercellular communication in cancer progression, we further analyzed cytokine signaling activity in the transcriptomic profile using CytoSig (26) to predict the effect of cytokines from the transcriptome-level signaling cascade.

### Differential expression and pathway enrichment analysis

The differentially expressed gene levels of samples in the high-CRD and low-CRD groups were calculated using the R package DESeq2 V1.30.1 (27). We set FDR< 0.05 and |log_2_FC| > 1 as the criteria for differentially expressed genes. Pathway enrichment analysis based on cancer hallmarks and the Kyoto Encyclopedia of Genes and Genomes (KEGG) signaling (28) pathway was conducted using the enricher function of the R package clusterProfile V3.18.1 (29).

### Immune and stromal infiltration

We used CIBERSORT (30) to determine the relative composition of immune cells in TCGA-LIHC samples based on the gene expression matrix. The overall immune and stromal infiltration levels were estimated using the R package Estimate V1.0.13 (31).

### Drug susceptibility and immunotherapy response analysis

We downloaded gene expression matrices and half-maximal inhibitory concentration (IC50) values of 805 cell lines treated with 198 drugs from the Genomics of Drug Sensitivity in Cancer (GDSC) database (32). Based on this training dataset, we used the R package oncoPredict V0.2 to build a ridge regression model and predict the response of each TCGA sample to each drug. The change in response of the high-risk and low-risk groups to each drug represented the difference in drug response between the two groups. Tumor immune infiltration data were downloaded from the Tumor Immune Dysfunction and Exclusion (TIDE) database (http://tide.dfci.harvard.edu/download/). Immunophenoscores (IPSs) were downloaded from the Cancer Immunome Atlas (https://tcia.at/home). Higher tumor-infiltrating cell exclusion scores and lower IPSs predicted poorer responses to immunotherapy.

### Mutation analysis

Somatic mutation data on the TCGA-LUAD samples were downloaded from the UCSC Xena browser (https://xenabrowser.net/). The mutational landscape and somatic mutation interactions of samples with high and low CRD levels were analyzed using maftools V2.6.05. Among the various types of mutations, Missense, Nonsense, Frame_Shift_Ins, Frame_Shift_Del, In_Frame_Ins, In_Frame_Del, Splice_Site, Translation_Start_Site, and Nonstop were considered nonsynonymous mutant variants. Silent and other types of mutations, including introns, 3’ untranslated regions, 5’ untranslated regions, and intergenic regions, were considered synonymous mutant variants. Synonymous mutations were also considered wild-types because they did not affect proteins. Frameshift and nonsynonymous mutant variants were encoded as truncating mutations, consistent with previous studies (33). Mutation proportions were assessed using one-sided Z-tests and two-sided χ^2^ tests. Statistical significance was set at p< 0.05.

### Prognostic model construction

To better apply CRD scores in clinical sample analysis and make it possible to evaluate patient prognosis, we used the LASSO-Cox regression model to screen potential genes significantly associated with prognosis based on the 206 CRGs that were significantly positively correlated with the tumor stage. After 1,000 permutations and cross-validation, 14 genes were screened. Linear combinations of these genes (based on their expression levels) were used to calculate the CRD scores of the HCC samples from each patient. The minimum criterion determined the regression coefficient. The final CRD risk score of each sample was calculated as follows:


$$\begin{array}{c} \text{CRD risk score }=\;0.112\times \text{ENO}1+\;0.077\times \text{STMN}1+\;0.108\\ \;\;\;\;\;\;\times \text{SLC}1\text{A}7+\;0.055\times \text{RAB}13+\;0.019\\ \;\;\;\;\;\;\times \text{SRPRB}+\;0.019\times \text{UGDH}-\;0.039\\ \;\;\;\;\;\;\times \text{RCAN}2+0.201\times \text{HILPDA}+\;0.042\\ \;\;\;\;\;\;\times \text{WEE}1+\;0.159\times \text{HSPA}8+\;0.022\\ \;\;\;\;\;\;\times \text{BAMBI}-\;0.383\times \text{P}2\text{RX}1-\;0.053\times \text{UBB}\\ \;\;\;\;\;\;+\;0.048\times \text{MAFG} \end{array}$$

We subsequently divided the samples into high-risk and low-risk groups based on the median CRD score. The statistical significance of overall survival (OS) was determined using the log-rank test and visualized as Kaplan–Meier curves using the R package survminer V0.4.9.

## Results

### Malignant cells with CRD correlate with disease phenotypes

**Figure 1:**
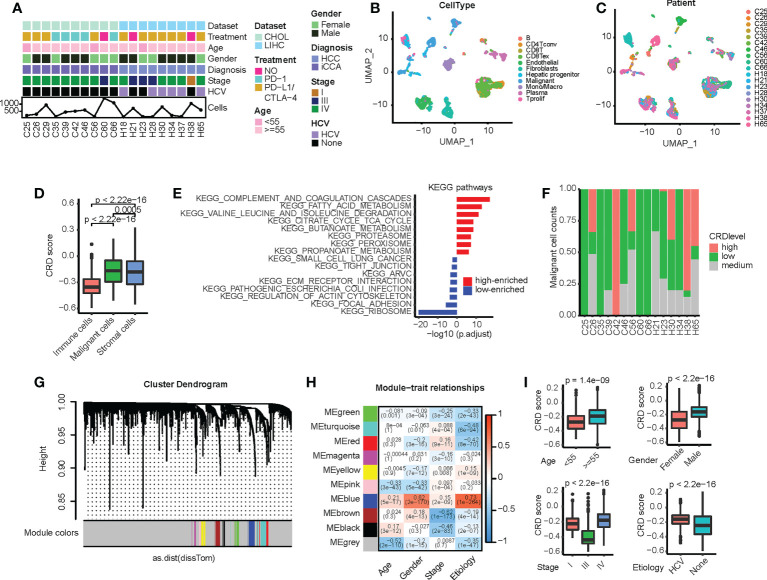
Circadian rhythm disruption (CRD) heterogeneity across different sample origins in malignant cells. **(A)** Clinical and molecular properties of liver cancer patient samples from the scRNA dataset. **(B)** Uniform manifold approximation and projection (UMAP) of malignant and non-malignant cells obtained from patients. **(C)** UMAP of all cells obtained from patients. **(D)** CRD score of cells calculated *via* single-sample gene set enrichment analysis. **(E)** Differences in pathway enrichment between high-CRD and low-CRD groups. **(F)** CRD score distribution across patients. **(G)** Division of CRD-related genes (CRGs) into multiple modules. **(H)** Correlation between different gene modules and clinical variables. **(I)** Correlation between CRD scores and clinical indicators (age, gender, cancer stage, and viral infection status).

Most patients in both single-cell transcriptome datasets received PD-1 or PD-L1/CTLA-4 monoclonal antibody therapy and predominantly exhibited stage IV liver cancer development (**Figure 1A**). The cell numbers identified for each patient ranges from 109 to 1, 416. The merged, regrouped, and annotated single-cell transcriptome datasets are shown in **Figures S1A, B**. As shown in **Figures 1B, C**, malignant cells differed significantly between patients, whereas immune cells were more consistent. The epithelial score of malignant cells was markedly higher than that of stromal and immune cells (**Figure S1C**), which is consistent with previous studies (34). Marker genes in different clusters were highly expressed in specific immune cells (**Figure S1D**), further supporting the accuracy of our clustering. Based on the collected CRGs (**Table S1**), the CRD score of malignant cells was significantly higher than that of other cells, indicating that the malignant cell cycle was more severely disordered (**Figure 1D**). We further divided the malignant cells into groups with high, medium, and low CRD scores (**Figure S1E**). We found that cells with a high CRD score were enriched in various metabolic signaling pathways. In contrast, cells with a low CRD score were significantly enriched in signaling pathways such as cell junctions (**Figure 1E**). Moreover, the CRD score of malignant cells varied widely between different samples (**Figure 1F**), indicating that liver cancer has high intratumoral heterogeneity.

We subsequently screened for CRGs associated with clinical factors. First, we used the WGCNA method to analyze the single-cell transcriptome matrix (**Figure S2A**) and divide the CRGs into multiple modules (**Figure 1G**). The relationship between core genes and clinical variables in each module was then calculated (**Figure 1H**), and genes significantly associated with these variables were identified (**Table S2**). We found that these genes were greatly enriched in tumorigenesis and development-related signaling pathways, such as epithelial-mesenchymal transition, MAPK signaling, P53 signaling, hypoxia, and cytokine receptor interaction (**Figure S2B**), suggesting that these CRD scores are closely related to the malignant progression of HCC. In addition, the CRD score was significantly correlated with clinical variables, such as age, sex, cancer stage, and the viral infection status of the samples (**Figure 1I**).

### CRD remodels cellular communication in the tumor microenvironment

**Figure 2:**
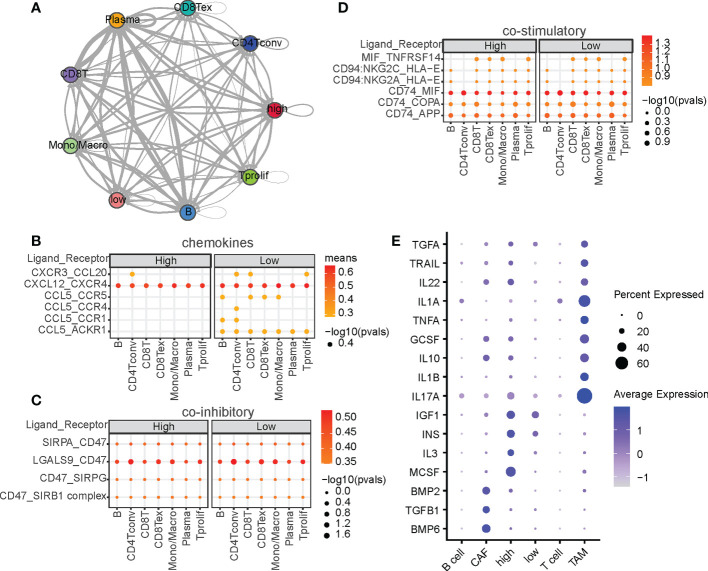
Intercellular ligand-receptors and cytokine‐related pathway network analysis. **(A)** Intercellular communication networks among malignant and non-malignant cells. Interactions between malignant cells and other cell subpopulations through ligan-receptors (LRs) are divided into **(B)** chemokines, **(C)** co-stimulatory factors, and **(D)** coinhibitory factors. P-values are indicated by circle size, and the average expression level of LRs is indicated by color. **(E)** Summary of signaling activities of representative cytokines in multiple cell types.

Cytokine interactions between immune cells and malignant cells in the tumor microenvironment (TME) have always been the focus of research in antitumor immunotherapy (35, 36). Owing to the significant enrichment of cytokine interaction signaling pathways reported in Section 3.1, we speculated that cells in the high-CRD and low-CRD groups and immune cells in the TME are also involved in other types of intercellular communication. We thus used the CellphoneDB to analyze intercellular interactions and construct an intercellular communication network (**Figure 2A**). Notably, among the chemokines, high-CRD and low-CRD groups exhibited distinct intercellular communication with immune cells (**Figure 2B**), including CCL20, CXCL12, and CCL5, all of which were affected by CRD. In the low-CRD group, the accumulation of CCL5/CCR5, CCL5/ACKR1, and CXCR3/CCL20 suggests the accumulation of CD8(+) T cells. These results indicate that the low-CRD group may have had a higher level of immune infiltration. In comparison, the high-CRD group may have had a lower level of immune infiltration and inconsistent immunotherapy responses. We also compared immune co-suppressive factors (**Figure 2C**) and immune co-stimulatory factors (**Figure 2D**). However, we did not find significant differences between the low-CRD and high-CRD groups. To our knowledge, in the tumor microenvironment, chemokines can be expressed in various types of cells, and their expression levels are high, while stimulatory factors and inhibitory factors are not much detectable, and their expression levels are also limited. Therefore, chemokine expression is more easily detected and differential chemokines are more easily identified, so it is easier for us to find differences in chemokines from the results.

Cytokines are critical for intercellular communication in cancer progression (37). To investigated whether CRDs regulate cytokine signaling activity, we calculated the cytokine activities at the single-cell level. As shown in **Figure 2E**, cytokines such as IL17A and IL1A were upregulated in TAM, and cytokines such as MCSF, IL3, IGF1, and INS were significantly upregulated in the high-CRD group. IL17A and IL1A are two well-known regulators of macrophages (38, 39), indicating the correctness of the cytokine activity we calculated. MCSF is the macrophage colony-stimulating factor that regulate the differentiation of myeloid lineage cells (40), and IL3 plays functional role on regulating the myofibroblastic differentiation (41). The diversity of CRD scores suggests differences in the TME composition—CRD may be a significant regulator of immune cell accumulation in HCC.

### CRD status correlates with tumor progression and the immune microenvironment

**Figure 3:**
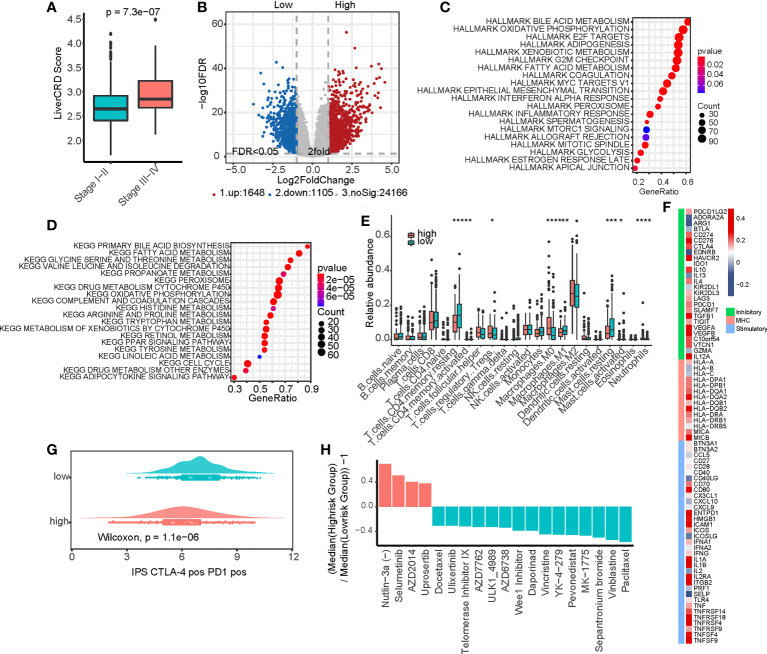
Functional analysis of the CRD enrichment (LiverCRD) score and liver hepatocellular carcinoma immune signature. **(A)** Differences in the distribution of LiverCRD scores at different TNM stages. **(B)** Differentially expressed genes between high-risk and low-risk subgroups. **(C)** Hallmark enrichment analysis of the distribution of LiverCRD) scores. **(D)** Kyoto Encyclopedia of Genes and Genomes enrichment analysis of LiverCRD score distribution. **(E)** Differences in immune cell distribution between high-risk and low-risk subgroups. ns means P > 0.05, * means P ≤ 0.05, ** means P ≤ 0.01, *** means P ≤ 0.001, **** means P ≤ 0.0001. **(F)** Relationship between LiverCRD scores and immune modulators. **(G)** Distribution of patient immunophenoscores under anti-CTLA-4 and anti-PD-1 treatment between high-CRD and low-CRD subgroups. **(H)** Differences in drug response between high-risk and low-risk subgroups.

Based on the CRGs related to clinical variables screened in Section 3.1, we used the ssGSEA method to evaluate the CRD status of the bulk samples. We defined the enrichment score as the LiverCRD score (**Table S3**), which significantly correlated with tumor stage (**Figures 3A**, **S3A**). We further divided the samples into high and low median scores and compared their gene expression and pathway activity levels (**Figure 3B** and **Table S4**). Similar to the single-cell transcriptome results in Section 3.1, in both the cancer hallmark and KEGG signaling pathway datasets, we observed the differential activation of many metabolism-related signaling pathways, such as bile acid and fatty acid metabolism. This coincided with the activation of many signals related to tumor development, such as the MYC signaling pathway, E2F target, oxidative phosphorylation, interferon response, and mTOR signaling pathway (**Figures 3C, D**).

These results suggest that CRD is likely associated with the remodeling of the immune microenvironment. Although the LiverCRD and immune/stromal infiltration scores estimated using the Estimate algorithm did not significantly correlate with each other (**Figure S3B**), the two groups exhibited significant differences in T cell and macrophage levels (**Figure 3E**). Many immune checkpoints were higher in the high-LiverCRD group (**Figure S4A**), and the expression of immune checkpoints, such as PD-1 and CTLA-4, was significantly positively correlated with the LiverCRD score (**Figure S4B).** Many immune-related molecules, such as MHC molecules, immune stimulators, and immune inhibitors, were regulated by CRD (**Figure 3F**). Based on immune cell exclusion and dysfunction levels downloaded from the TIDE database, the high-LiverCRD group had a higher level of immune cell rejection (**Figure S4C**). Our predictions suggested that the high-LiverCRD group had worse response to immunotherapy than the low-LiverCRD group (**Figures 3G**, **S4D**). These results reflect those of a previous single-cell transcriptome analysis, which showed that the immune microenvironment of the high-CRD group had a lower level of immune infiltration and lower CD8(+) T cell content than the low-CRD group (42). Based on the gene expression matrix and IC50 value of the GDSC database, we constructed a ridge regression model to predict the drug response of TCGA samples (**Table S5**). The high-LiverCRD group responded better to MDM2, MEK, and mTOR inhibitors than other drugs (**Figure 3H**), reflecting the higher activity of these pathways in the high-CRD group in the previous analysis.

### CRD status affects the mutational landscape

**Figure 4:**
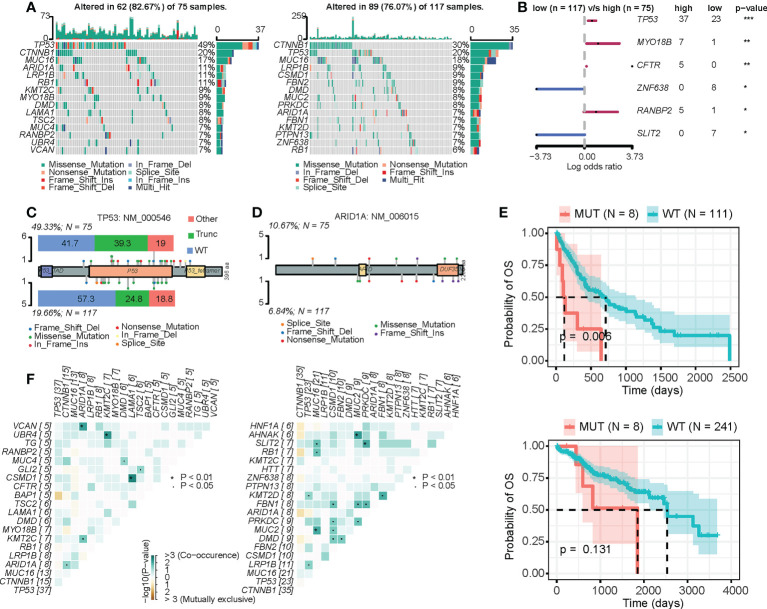
Mutation differences between the high-CRD and low-CRD groups. **(A)** Mutational landscapes of high-CRD and low-CRD groups. **(B)** Forest plot of differentially mutated genes in high-CRD and low-CRD patients. **(C)** Lollipop chart of TP53 protein mutation sites. **(D)** Lollipop chart of ARID1A protein mutation sites. **(E)** Kaplan–Meier survival curves for patients in high-LiverCRD and low-LiverCRD groups in relation to ARID1A mutation. **(F)** Mutation co-occurrence and difference in mutually exclusive patterns between high-CRD and low-CRD groups.

The precise regulation of the cell cycle relies on a series of regulatory molecules, among which mutations are particularly critical (43). We thus analyzed the mutational landscape of the high-CRD and low-CRD groups (**Figure 4A**). As shown in **Figure 4B**, TP53 and MYO18B more frequently mutated in the high-CRD group than the low-CRD group. In addition, TP53 mutations in the high-CRD group were more likely to be enriched *via* truncating mutations that affect protein function. In contrast, the number of TP53 mutations in the low-CRD group that did not affect protein function was relatively high (**Figure 4C**), suggesting that CRD may be regulated by TP53 mutation status. Notably, although ARID1A mutation levels and types were not significantly different between the two groups (**Figure 4D**), ARID1A mutations were associated with lower patient survival rates in the high-CRD group than in the low-CRD group (**Figure 4E**), suggesting that ARID1A mutations in the former were affected by factors performing different functions. We also compared the somatic mutation interactions between the two groups. Although the two groups of mutations generally co-occurred, the specific crosstalk differed between them (**Figure 4F**). The ARID1A/VCAN, KMT2C/UBR4, and LAMA1/CSMD1 pair mutations co-occurred in the high-CRD group. The MUC2/AHNAK, PRKDC/SLIT2, and MUC16/MUC2 pairs of mutations co-occurred in the low-CRD group.

### Fourteen-gene signature predicts survival in patients with liver cancer

**Figure 5:**
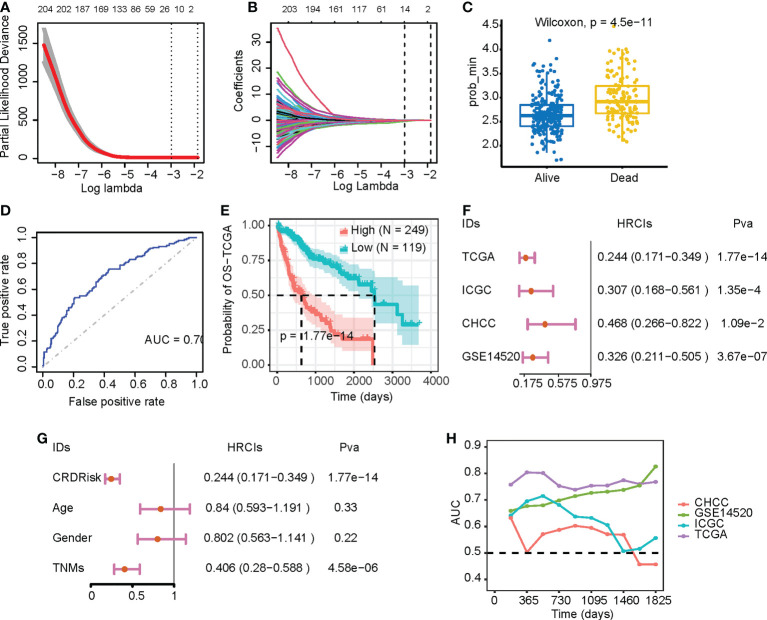
Evaluation of the prognostic value of CRDs. **(A, B)** Parameters of the LASSO-Cox regression model after 1,000 permutations and cross-validation. **(C)** CRD risk scores in relation to patient survival. **(D)** Receiver operating characteristic curves of patients in the high-risk and low-risk groups of the Cancer Genome Atlas (TCGA) dataset. **(E)** Kaplan–Meier survival curves of patients in the high-risk and low-risk groups of the TCGA dataset. **(F)** Forest plot of the LASSO-Cox analysis of the TCGA and three validation datasets. **(G)** Forest plot of the LASSO-Cox analysis of the TCGA dataset based on CRD risk scores and clinical variables. **(H)** Time-dependent area-under-the-curve value of the TCGA and three validation datasets.

After 1,000 permutations and cross-validation, we determined the parameters of the LASSO-Cox regression model (**Figure 5A**). The model was constructed using 14 genes and calculated their respective regression coefficients (**Figure 5B** and **Table S6**). As shown in **Figure 5C**, patients who died had significantly higher CRD risk scores, indicating that CRD risk significantly affected patient survival (**Figure 5D**). As shown in **Figure 5E**, the median survival times of the high-risk and low-risk groups were approximately 800 and 2,500 days, respectively. We verified the robustness of the high-risk scores using three independent datasets. As shown in **Figure 5F**, the CRD risk scores of different datasets significantly correlated with the hazard ratio, and their respective patient survival curves also differed significantly (**Figure S5**). Although these datasets were sequenced by various countries, the survival analysis was performed within a single dataset, and survival differences were consistent across datasets. These results suggest that the CRD risk score is a robust indicator of survival in HCC patients.

Compared with clinical factors, the CRD score could assess patient risk similarly to tumor stage (**Figure 5G**). Time-dependent receiver operating characteristic curve analysis also indicated that the CRD risk score was effective in assessing patients 1-, 3-, and the effect was stable in the 5-year survival state (**Figure 5H**). In addition, we constructed a nomogram for multivariate analysis, showing that the CRD risk score predicted overall survival (**Figure S6A**). Calibration curves showed that the CRD risk score did not deviate significantly from the actual value in predicting one-, three-, and five-year patient survival (**Figures S6B–D**, respectively). These results suggest that the CRD risk score is a suitable indicator for monitoring HCC patient survival.

## Discussion

Circadian rhythm disruptions affect the metabolism of tumor cells and control the progression of tumor development by interacting with non-clock transcription factors, ultimately affecting cell differentiation and proliferation (9–11). As the circadian clock is intimately involved in regulating the metabolism in peripheral tissues, and because most metabolite levels in the liver and serum are controlled in a cyclical manner (44), the regulation of CRD and cancer metabolism has become a research hotspot. Meta-analysis has revealed widespread misexpression of clock genes in multiple human cancers (14). Notably, the abnormal expression of clock genes appears to correlate strongly with the stage or aggressiveness of various cancers. In this study, we employed a computational method to calculate and assess intratumoral CRD in liver cancer cells using a single-cell transcriptomic dataset. CRD scores predicted tumor responses to ICB therapy and can guide targeted strategies for chemotherapy. Furthermore, a LASSO-Cox regression model based on 14 CRD genes was used to predict overall patient survival across multiple datasets. Through systematic analysis, we aimed to determine the role of circadian rhythm disruption in tumor progression and prognosis. Targeted drug prediction analysis can provide new therapeutic strategies for combination antitumor therapy.

Our results suggest that circadian rhythm disruptions regulate the activity of various metabolic pathways, such as bile acid and fatty acid metabolism. We also observed abnormal perturbations of the MYC and the p53 signaling pathways. The MYC protein has been reported to play an essential role in regulating rhythmic metabolism in cultured U2OS human osteosarcoma cells (12). The oncogenic potential of MYC is due to its ability to activate gene expression related to cell survival and proliferation (45). Ectopic MYC expression disrupts circadian gene expression (46). The MYC protein activates the negative transcriptional arm of the circadian clock and stimulates metabolic sensing pathways such as AMPK, ultimately leading to increased glucose and glutamine consumption (12). p53 is a transcription factor that regulates cell cycle arrest and apoptosis through activation (47). Important cell-cycle checkpoint functions are provided by p53-mediated arrest. p53 protects chromosomal integrity and increases cell longevity by halting cells in the G1 phase and enabling time for the repair of possibly lethal double-strand breaks. p53 also regulates a series of genes involved in DNA recombination and repair (48). After ionizing irradiation, p53-null cells display impairments in the repair of double-strand breaks in heterochromatin and have worse long-term survival (49). These results illustrate that oncoproteins disrupt circadian function and subsequently affect cellular functions.

We found that cells with a high CRD score were enriched in various metabolic signaling pathways. In contrast, cells with a low CRD score were significantly enriched in signaling pathways such as cell junctions. In the low-CRD group, the accumulation of CCL5/CCR5, CCL5/ACKR1, and CXCR3/CCL20 suggests the accumulation of CD8(+) T cells, which indicated that the low-CRD group may have had a higher level of immune infiltration. Interestingly, metabolic reprogramming occurs frequently in cancer cells, which induce a reprograming of tumor microenvironment (50). The significantly enriched pathways in high-CRD group, such as fatty acid metabolism and TCA cycle, were proved to induce a immunosuppression environment. These results provided a potential insight of circadian rhythm disruption in regulating tumor microenvironment reprogramming. This study also provided further evidence suggesting that CRD may inhibit T cell aggregation by inhibiting the expression of CCL5 (51). This chemokine regulates T cell entry into the TME and may thus play a crucial role in T cell infiltration in solid tumors (52). This also explains why immunotherapy in the high-CRD group was not viable. Our subsequent analysis provided potential chemotherapy regimens for this type of tumor, including MDM2, MEK, and mTOR inhibitors. Notably, mTOR inhibition slows the circadian clock and suppresses clock oscillations, whereas mTOR activation accelerates and enhances clock oscillations (53). Lastly, MEK inhibitors are critical for regulating circadian clock activity (54).

We also found that ARID1A mutations lead to different survival outcomes in different subgroups, which is indeed an interesting discovery. ARID1A mutations were found in over 30% of various cancer types (55, 56). Previous studies found that the majority of ARID1A mutations are inactivating mutations, which lead to loss of ARID1A expression (57, 58). In ARID1A-deficient tumors, the cell cycle checkpoint proteins ATM/Chk2 axis is inhibited (59), thereby alters the expression of key cell cycle regulators (60). Therefore, we speculate that ARID1A mutations may exacerbate circadian rhythm disruption, which lead to different survival outcomes. What’s more, a fourteen-gene based LASSO-Cox regression model was used to predict overall patient survival across multiple datasets. The most correlated gene hypoxia-inducible lipid droplet-associated (HILPDA) is differentially expressed in various tumors (61). HILPDA could act as an oncogenic factor modulating cell cycle pathway, which represent a novel biomarker of tumorigenesis (62).

However, limitations still exist. The data we used all rely on public datasets, and it is necessary to measure our own sequencing data for analysis in the future. Furthermore, our conclusions are mainly obtained by bioinformatics analysis and lack critical experimental validation. Although we performed cross-validation on multiple datasets to evaluate the robustness of the model, immunohistochemical validation of the expression of these modeled genes was necessary. Finally, we expounded the function and clinical significance of CRD in liver cancer, but the molecular mechanism is still lacking. We need to carry out exhaustive verification of our analysis results in the future to clarify the biological mechanisms of CRD in liver cancer.

In conclusion, we systematically analyzed the carcinogenesis mechanism of CRD and assessed the intratumoral CRD level of liver cancer based on single-cell transcriptome data. A LASSO-Cox regression model constructed based on 14 genes was accurately predicted the overall survival of patients in multiple datasets, suggesting that CRD can potentially be used as a prognostic indicator. These results form a basis for regulating tumor progression and guiding potential targeting strategies.

## Data availability statement

Publicly available datasets were analyzed in this study. This data can be found here: The gene expression matrix of the Cancer Genome Atlas Liver Hepatocellular Carcinoma (TCGA-LIHC) dataset was downloaded from the University of California, Santa Cruz (UCSC) Xena browser (https://xenabrowser.net/). Transcriptomic and clinical data from Chinese HCC patients with HBV infection were downloaded from the National Omics Data Encyclopedia database (https://www.biosino.org/node/) using the accession number OEP000321. The LIRI-JP dataset was downloaded from the International Cancer Genome Consortium Data Portal (https://dcc.icgc.org/). Gene expression microarray data and detailed clinical information on GSE14520 were downloaded from the Gene Expression Omnibus database (https://www.ncbi.nlm.nih.gov/geo/).

## Author contributions

YS and R-QW designed and supervised the experiments. R-QW performed experiments and data analysis. WC and JC contributed to data analysis and predictor development. R-QW wrote the manuscript with contributions from all the authors. All authors contributed to the article and approved the submitted version.

## Funding

This study was supported by the Guangdong Basic and Applied Basic Research Foundation [2020A1515110057], the Xiangshan Talented Scientific Research Foundation of Zhuhai People’s Hospital [2020XSYC-07; 2021XSYC-02], the National Natural Science Foundation of China (32100561, 82102163).

## Acknowledgments

We would like to thank Editage (https://www.editage.com) for English language editing.

## Conflict of interest

The authors declare that the research was conducted in the absence of any commercial or financial relationships that could be construed as a potential conflict of interest.

## Publisher’s note

All claims expressed in this article are solely those of the authors and do not necessarily represent those of their affiliated organizations, or those of the publisher, the editors and the reviewers. Any product that may be evaluated in this article, or claim that may be made by its manufacturer, is not guaranteed or endorsed by the publisher.

## Supplementary material

Supplementary Figure 1 Overview of the integrated liver cancer scRNA transcriptome dataset. **(A)** Principal components determination for scRNA transcriptome data clustering. **(B)** Uniform manifold approximation and projection (UMAP) of all cells from the two groups. **(C)** Distribution of epithelial scores among malignant cells, immune cells, and stromal cells. **(D)** Marker genes expression in different cell groups. **(E)** UMAP of the Circadian rhythm disruption (CRD) score distribution in malignant cells across the patients

Supplementary Figure 2
**(A)** Soft threshold determination for weighted correlation network analysis (WGCNA). **(B)** Kyoto Encyclopedia of Genes and Genomes and cancer hallmark pathways enrichment analysis of the distribution of CRD scores in malignant cells.

Supplementary Figure 3 Relationship between CRD enrichment (LiverCRD) score and clinical variables. **(A)** Differences in the distribution of LiverCRD scores between different clinical variables. **(B)**

Supplementary Figure 4 LiverCRD score associated with the immune checkpoint therapy response. **(A)** Differences in the distribution of immune checkpoints between high-risk and low-risk subgroups. **(B)** Relationship between LiverCRD score and CD274/CTLA-4. **(C)** Relationship between LiverCRD score and immune cell exclusion/dysfunction level. **(D)** Distribution of patient immunophenoscores under anti-PD-1 and anti-CTLA-4 treatment between high-risk and low-risk subgroups.

Supplementary Figure 5 Kaplan–Meier survival curves of patients in the high-risk and low-risk groups of the three validation datasets.

Supplementary Figure 6 Nomogram and calibration analysis. **(A)** Nomogram of age, gender, TNM stage, and CRD risk score in the prediction of one-, three-, and five-year probability of overall survival (OS). Calibration plot for the evaluation of the nomogram in predicting **(B)** one-year, **(C)** three-year, and **(D)** five-year OS probability.

Supplementary Table 1 Circadian rhythm disruption (CRD)-related gene list collected from the CircaDB database.

Supplementary Table 2 Genes across all modules that were significantly associated with clinical variables.

Supplementary Table 3 LiverCRD score of the Cancer Genome Atlas dataset calculated based on single-sample gene set enrichment analysis.

Supplementary Table 4 Differentially expressed genes between low-LiverCRD and high-LiverCRD groups.

Supplementary Table 5 Differences in drug response between low-LiverCRD and high-LiverCRD groups.

Supplementary Table 6 The 14 genes used to construct the LASSO-Cox regression model and their respective regression coefficients.

## Abbreviations

HBV, hepatitis B virus; HCV, hepatitis C virus; Treg, regulatory T; CRD, circadian rhythm disruptions; TFs, transcription factors; ROR, retinoic acid receptor-related orphan receptor; RREs, ROR response elements; CRGs, CRD-related genes; KEGG, Kyoto encyclopedia of genes and genomes; IC50, the 50% inhibiting concentration; GDSC, Genomics of Drug Sensitivity in Cancer; TIDE, tumor immune dysfunction and exclusion; IPS, Immunophenoscore; TCIA, The Cancer Immunome Atlas.
