# Supplementary figures and images for: Integrative analysis indicates the prognostic value of circadian rhythm disruption in liver cancer: Potential for therapeutic targeting
Source: Front Immunol. 2022 Nov 21;13:1011264. doi: 10.3389/fimmu.2022.1011264 (PMC9769576; doi:10.3389/fimmu.2022.1011264)

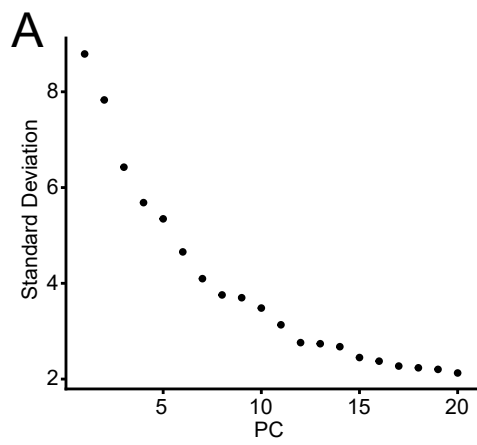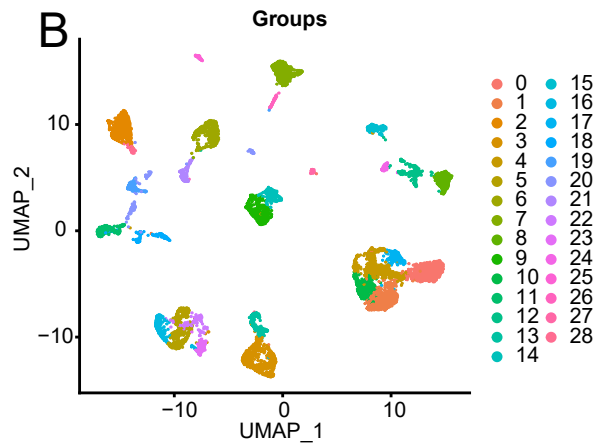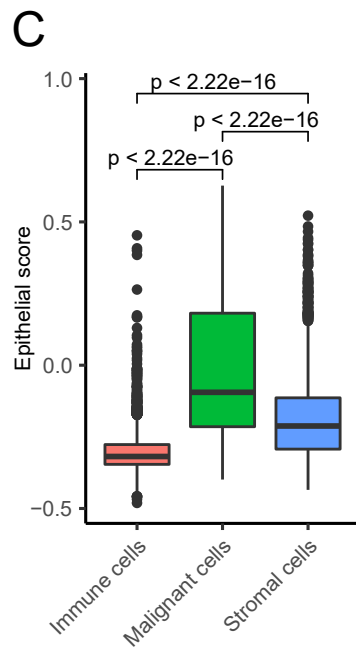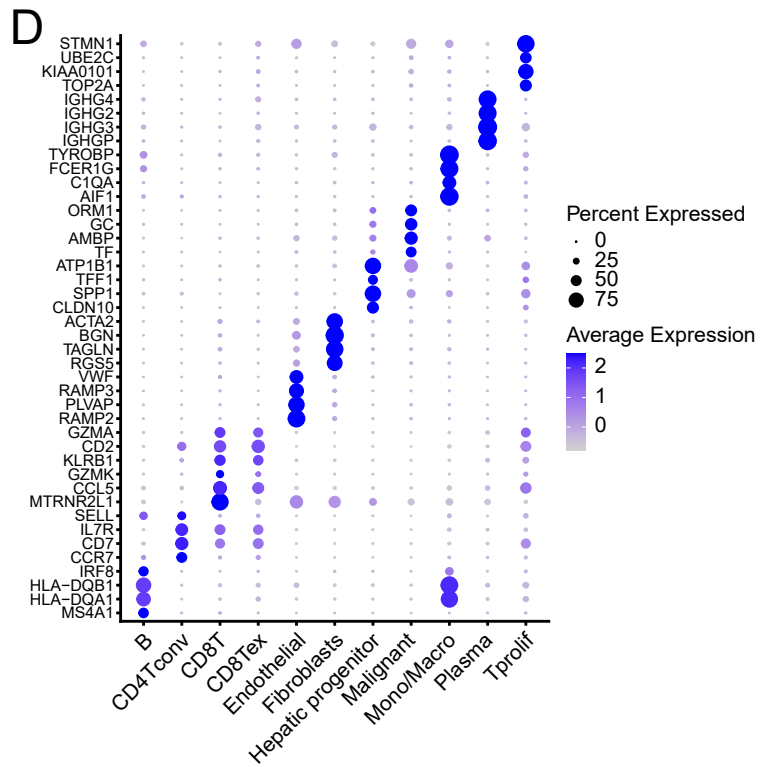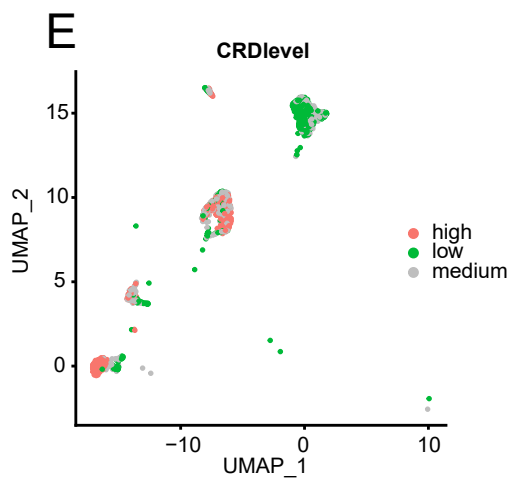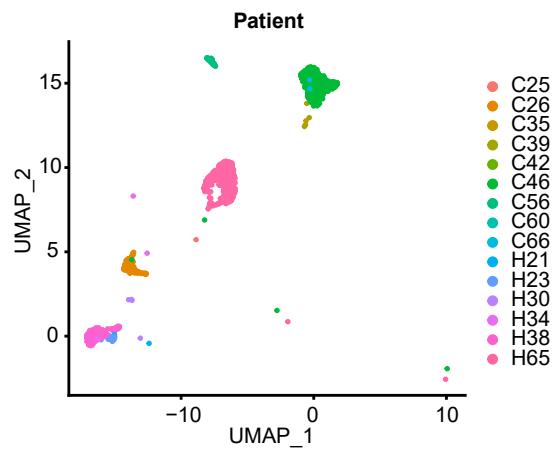

Supplement: Supplementary Figure 1 — Overview of the integrated liver cancer scRNA transcriptome dataset. (A) Principal components determination for scRNA transcriptome data clustering. (B) Uniform manifold approximation and projection (UMAP) of all cells from the two groups. (C) Distribution of epithelial scores among malignant cells, immune cells, and stromal cells. (D) Marker genes expression in different cell groups. (E) UMAP of the Circadian rhythm disruption (CRD) score distribution in malignant cells across the patients [file Image_1.pdf]

A

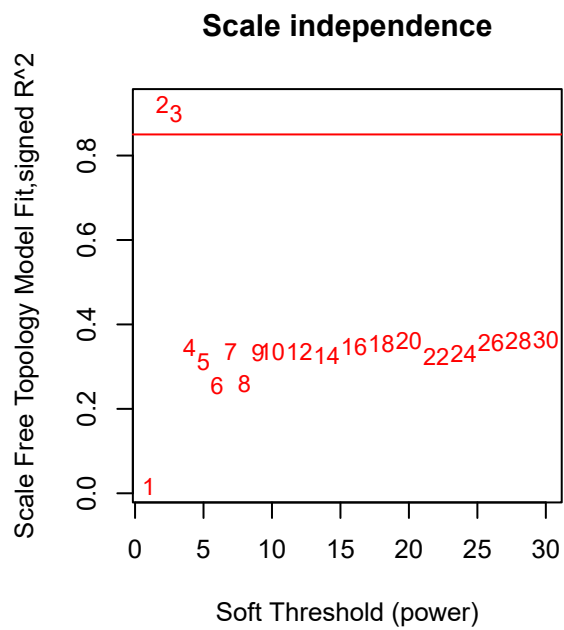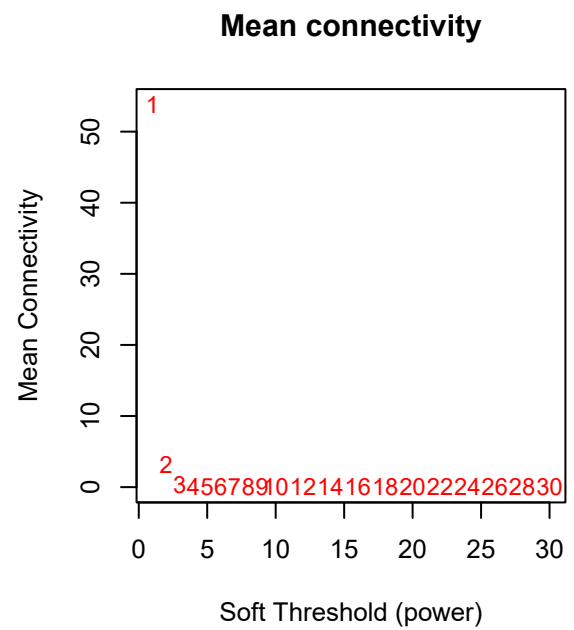

B

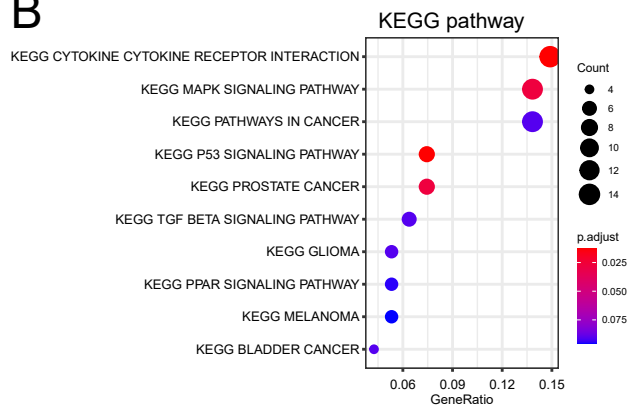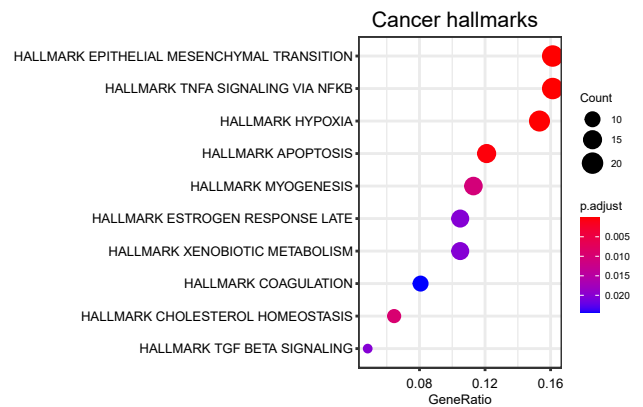

Supplement: Supplementary Figure 2 — (A) Soft threshold determination for weighted correlation network analysis (WGCNA). (B) Kyoto Encyclopedia of Genes and Genomes and cancer hallmark pathways enrichment analysis of the distribution of CRD scores in malignant cells. [file Image_2.pdf]

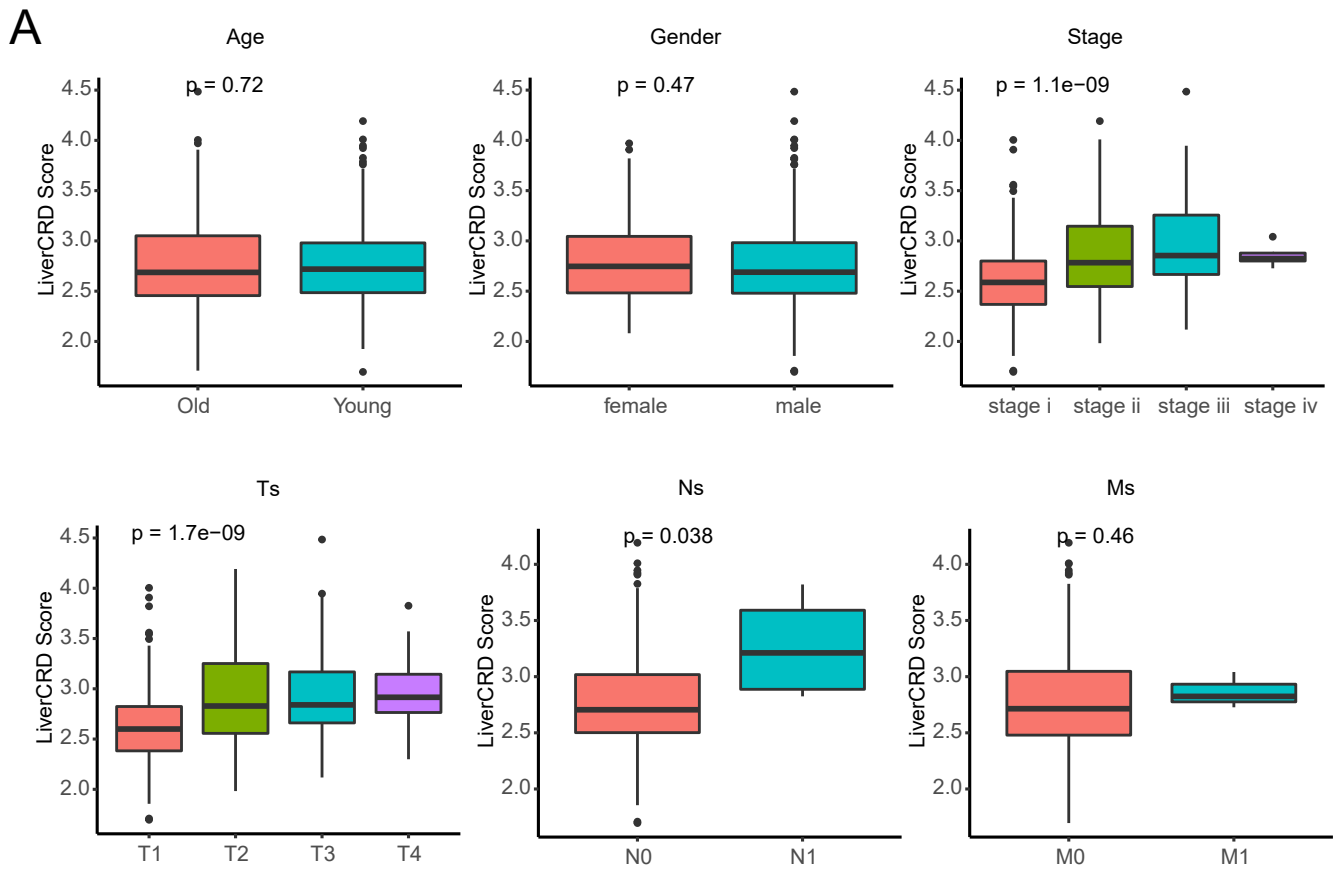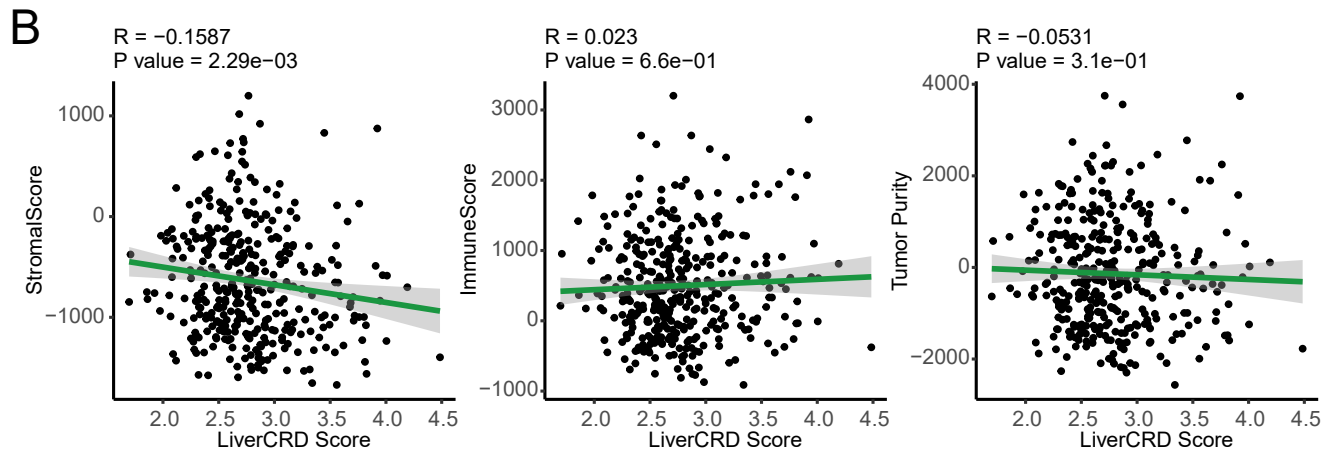

Supplement: Supplementary Figure 3 — Relationship between CRD enrichment (LiverCRD) score and clinical variables. (A) Differences in the distribution of LiverCRD scores between different clinical variables. (B) [file Image_3.pdf]

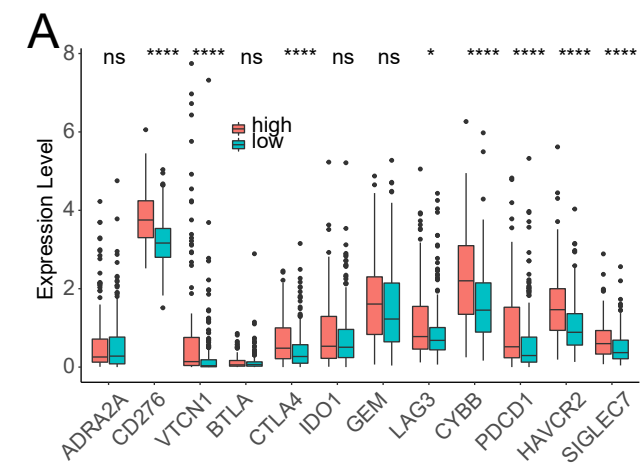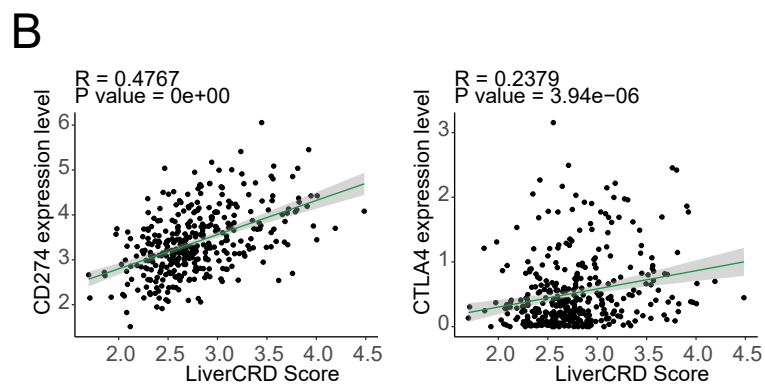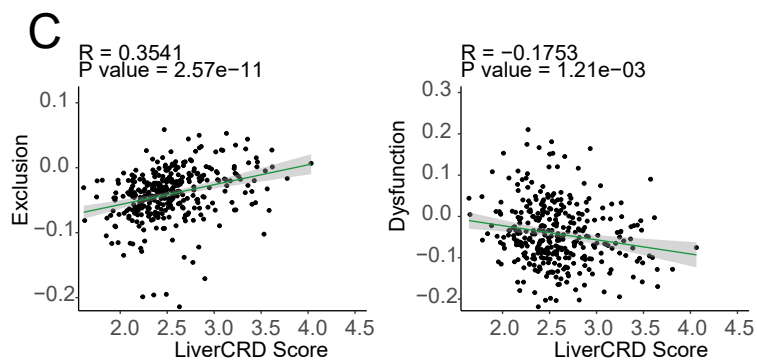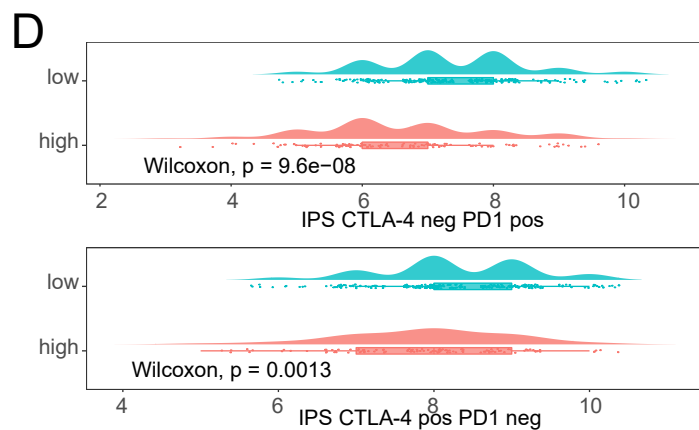

Supplement: Supplementary Figure 4 — LiverCRD score associated with the immune checkpoint therapy response. (A) Differences in the distribution of immune checkpoints between high-risk and low-risk subgroups. (B) Relationship between LiverCRD score and CD274/CTLA-4. (C) Relationship between LiverCRD score and immune cell exclusion/dysfunction level. (D) Distribution of patient immunophenoscores under anti-PD-1 and anti-CTLA-4 treatment between high-risk and low-risk subgroups. [file Image_4.pdf]

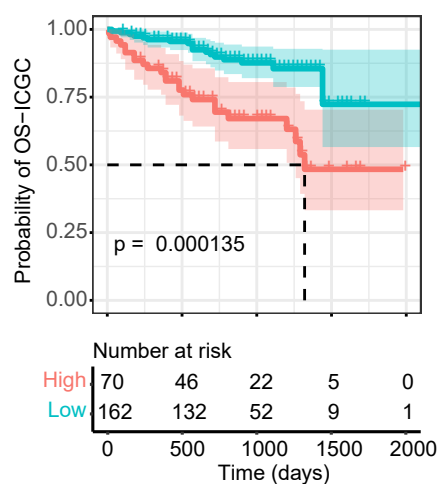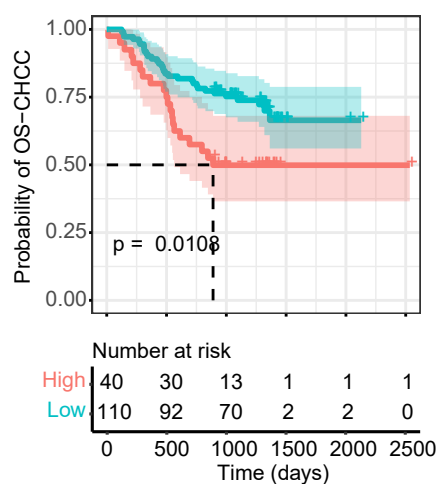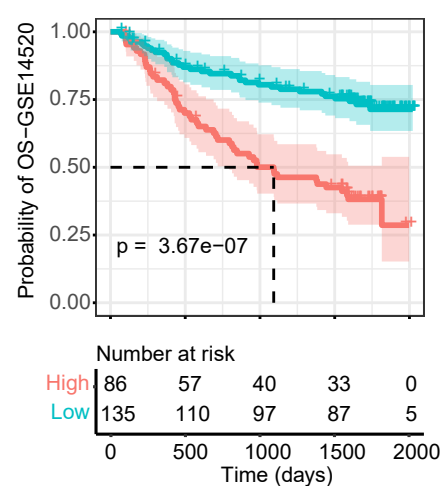

Supplement: Supplementary Figure 5 — Kaplan–Meier survival curves of patients in the high-risk and low-risk groups of the three validation datasets. [file Image_5.pdf]

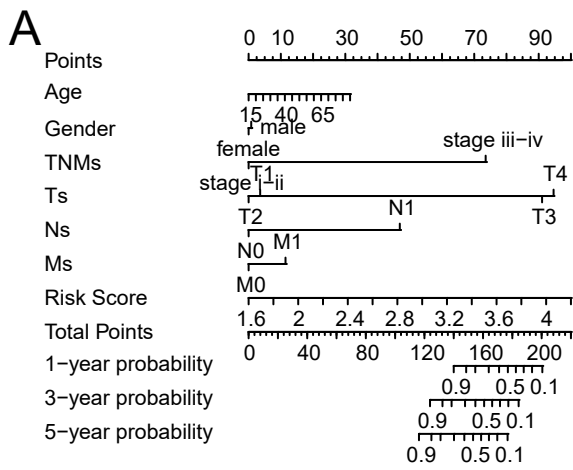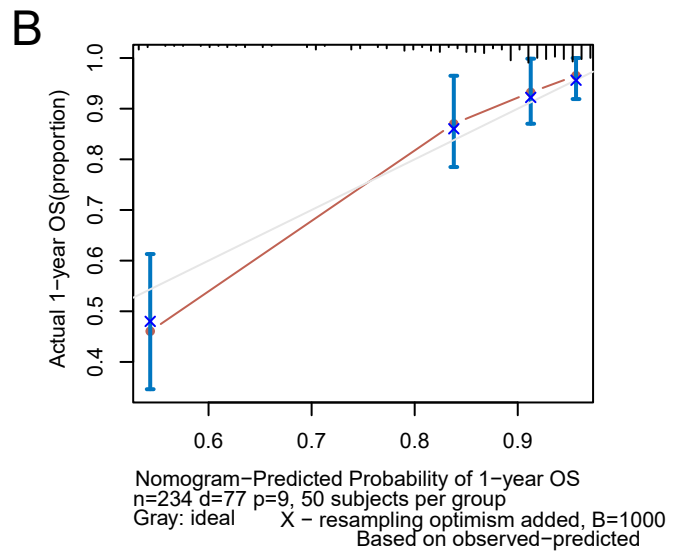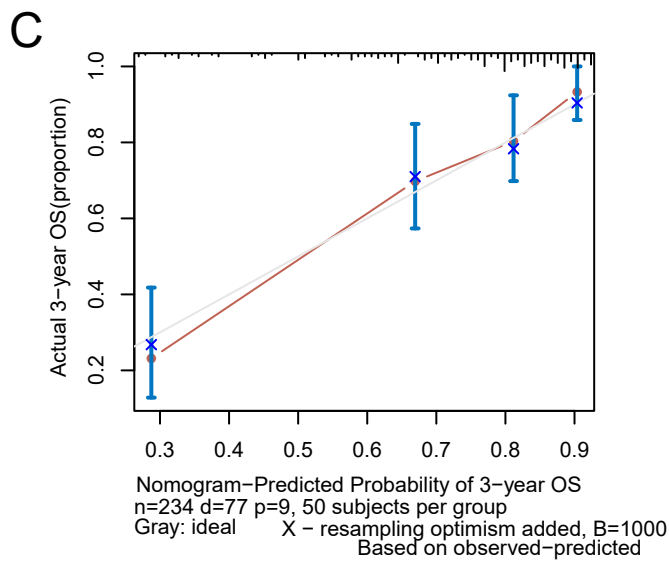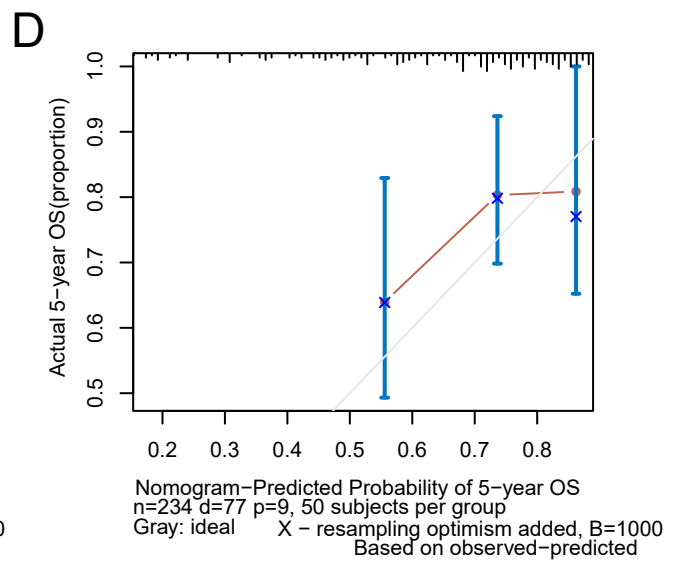

Supplement: Supplementary Figure 6 — Nomogram and calibration analysis. (A) Nomogram of age, gender, TNM stage, and CRD risk score in the prediction of one-, three-, and five-year probability of overall survival (OS). Calibration plot for the evaluation of the nomogram in predicting (B) one-year, (C) three-year, and (D) five-year OS probability. [file Image_6.pdf]
